# Accuracy of clinical predictions of prognosis at the end-of-life: evidence from routinely collected data in urgent care records

**DOI:** 10.1186/s12904-023-01155-y

**Published:** 2023-04-26

**Authors:** M. Orlovic, J. Droney, V. Vickerstaff, J. Rosling, A. Bearne, M. Powell, J. Riley, P. McFarlane, J. Koffman, P. Stone

**Affiliations:** 1 Royal Marsden NHS Foundation Trust, London, SW3 6JJ United Kingdom; 2 Imperial College London, London, United Kingdom; 3 Marie Curie Palliative Care Research Department, Division of Psychiatry, University College London, London, United Kingdom; 4 Hull York Medical School, Wolfson Palliative Care Research Centre, University of York, York, United Kingdom

**Keywords:** Prognosis, Death, Neoplasms, Palliative care, Dementia, Heart diseases, Lung diseases, Advance care planning

## Abstract

**Background:**

The accuracy of prognostication has important implications for patients, families, and health services since it may be linked to clinical decision-making, patient experience and outcomes and resource allocation. Study aim is to evaluate the accuracy of temporal predictions of survival in patients with cancer, dementia, heart, or respiratory disease.

**Methods:**

Accuracy of clinical prediction was evaluated using retrospective, observational cohort study of 98,187 individuals with a Coordinate My Care record, the Electronic Palliative Care Coordination System serving London, 2010–2020. The survival times of patients were summarised using median and interquartile ranges. Kaplan Meier survival curves were created to describe and compare survival across prognostic categories and disease trajectories. The extent of agreement between estimated and actual prognosis was quantified using linear weighted Kappa statistic.

**Results:**

Overall, 3% were predicted to live “days”; 13% “weeks”; 28% “months”; and 56% “year/years”. The agreement between estimated and actual prognosis using linear weighted Kappa statistic was highest for patients with dementia/frailty (0.75) and cancer (0.73). Clinicians’ estimates were able to discriminate (log-rank *p* < 0.001) between groups of patients with differing survival prospects. Across all disease groups, the accuracy of survival estimates was high for patients who were likely to live for fewer than 14 days (74% accuracy) or for more than one year (83% accuracy), but less accurate at predicting survival of “weeks” or “months” (32% accuracy).

**Conclusion:**

Clinicians are good at identifying individuals who will die imminently and those who will live for much longer. The accuracy of prognostication for these time frames differs across major disease categories, but remains acceptable even in non-cancer patients, including patients with dementia. Advance Care Planning and timely access to palliative care based on individual patient needs may be beneficial for those where there is significant prognostic uncertainty; those who are neither imminently dying nor expected to live for “years”.

**Supplementary Information:**

The online version contains supplementary material available at 10.1186/s12904-023-01155-y.

## Introduction

Clinicians are often required to estimate the survival of patients with advanced conditions [1]. Survival prognostication has important implications for patients, families and health care resource utilisation. Prognoses can influence clinical decisions about care pathways and treatment options, family expectations and psychological burden [2]. For patients with advanced disease, prognosis may be used to inform triage decisions, either for appropriate intensive hospital care or for timely access to community and end-of-life care resources. Failure to recognise when patients are approaching the end of life may contribute to more intensive, but ultimately futile, care [3]. Furthermore, inaccurate predictions may result in a delay in introducing interventions that could otherwise improve the quality of life and support for patients and their families [4]. Accurate prognostication can help patients to make plans and achieve goal-concordant care.

The World Health Organisation estimates that each year more than 40 million people require palliative care globally [5]. The need for palliative care is increasing, especially in developed countries due to an ageing population and greater numbers of patients living with complex comorbidities [6, 7]. Improved identification of patients who are approaching end-of-life may better inform patient care decisions as well as system-wide approaches and policies regarding the equitable provision and access to palliative and end-of-life care [8].

Survival prognostication is challenging [9] and clinical predictions tend to be inaccurate [10, 11]. Studies suggest that clinicians are often overly optimistic and unreliable in their estimates [12, 13]. Several prognostic tools or scoring systems exist but none has consistently displayed superiority against clinician predictions of survival [14–16].

Temporal predictions of survival relate to estimates about how long a patient has left to live [17, 18]. These can either be expressed as a continuous variable (e.g., a specific number of days, weeks, or months before death), or using discrete categories (e.g., between 1–2 weeks, etc.). Previous studies evaluating temporal predictions have been relatively small with samples sizes in the hundreds or few thousands [18].

Most studies examining prognostic accuracy have focused on hospital and hospice/palliative care patients, and those with cancer diagnoses. Prognostication appears to be even more inaccurate in patients with a non-cancer diagnosis [12, 19] but there have been few robust studies in this area. As the predicted trajectories of disease vary according to diagnostic category [20], a better understanding of prognostic accuracy in cohorts of patients according to diagnosis is needed to inform clinical training and appropriate application of prognostic estimates in clinical care.

This study aimed to evaluate clinicians’ accuracy of temporal predictions of survival for both cancer and non-cancer patients using routinely collected data from an Electronic Palliative Care Coordination System; an urgent clinical record system where information relevant to the delivery of a patient’s care can be recorded and accessed by health care professionals to improve coordination of care for patients, especially for those nearing the end of life [21, 22].

## Methods

### Setting and data source

The study used anonymised data routinely collected as part of Coordinate My Care, the largest Electronic Palliative Care Coordination System in UK, serving London 2010 -2022 [22]. Coordinate My Care is designed to facilitate patients and their clinicians in the making of advance care decisions and to share them digitally in real-time with relevant healthcare professionals involved in patients’ care. Coordinate My Care includes clinical information; patients’ preferences about end-of-life care such as their “preferred place of care”, “preferred place of death”; ceiling of treatment and resuscitation decisions. Since 2017, Coordinate My Care plans have contained a mandatory field that requires a temporal clinical prediction of survival. Clinicians are asked to provide a “likely prognosis” using a predetermined list of outcomes including the following categories: “days”, “weeks, “months”, “year”, “years” and “undefined”.

Patients consent to the use of their anonymised information for research at the time of consenting to the creation of a Coordinate My Care record. This service evaluation was approved by the Royal Marsden Committee for Clinical Research (1002, approved 14/09/2020). This study was co-produced with input from patients and members of the public and supported through a Biomedical Research Centre at the Royal Marsden and Institute for Cancer Research grant for Patient and Public Involvement.

### Sample

We included all individuals aged 18 or older with a Coordinate My Care plan registered between January 2010 and December 2020. The Coordinate My Care platform is connected to the NHS Spine, a digital NHS information exchange platform via which recorded dates of death are updated. Anonymised data were extracted in April 2021.

### Analyses

Stata (Version 14) was used for analyses. Demographics and clinical details were summarised for the study population and stratified according to whether the patient was alive or dead at the time of analysis. The overall cohort was also stratified into those with and without (missing) a defined survival estimate. Records containing a prognosis estimate were included in the analysis of accuracy (“Full prognosis cohort”). Records without a defined prognosis estimate were examined for differences compared to those with a documented prognosis.

Because the temporal estimates that clinicians used to make their prognostic estimates lacked clear boundaries (e.g., “weeks” is not precisely defined on the Coordinate My Care record) it was not possible to analyse the accuracy of predictions without making some assumptions about the boundaries of these categories. We defined a prognosis of “days” to mean 0–13 days, on the basis that if a patient survived for 14 days or more then they had already survived for “weeks”. Using similar logic, we defined “weeks” to mean ≥ 14 days and < 60 days (i.e., fewer than two months); “months” to mean ≥ 60 days and < 365 days (i.e., less than one year); and “years” to mean more than one year. As end-of-life care is typically focussed on patients in their last year of life, the fields “Year” and “Years” were combined to create a cohort of patients who had a longer prognosis. As well as having some face-validity, these boundary limits are like those used to define “days”; “weeks”, “months” and “years” in the well-validated Prognosis in Palliative care Study (PiPS) predictor models [9, 23, 24].

When more than one prognosis was recorded, the initial prognostic estimate was used for analysis. Survival was calculated as the number of days between the date of the initial survival prediction and the date of death if recorded. Survival predictions (“days”, “weeks”, “months”, “year/years”) were compared to individuals’ actual survival for the total study population and for groups of patients hypothesised to have distinct end-of-life illness trajectories [20]: cancer, dementia and frailty, heart disease and respiratory disease.

The accuracy of clinical prognostic estimates was defined as the frequency with which the clinician selected the correct survival category and was described as a percentage of the total number of estimates [9]. The extent of agreement between estimated and actual prognosis was quantified using linear weighted Kappa statistic. Kappa denotes the agreement between prognostic estimate and survival and considers that some prognoses could be expected to be correct purely based on chance. The kappa statistic result can be interpreted as follows: values ≤ 0 indicate no agreement; 0.01–0.20 none to slight; 0.21–0.40 fair; 0.41– 0.60 moderate; 0.61–0.80 substantial; and 0.81–1.00 as almost perfect agreement [25]. The survival times of patients were summarised using median and interquartile (IQ) ranges. Kaplan Meier survival curves were created to describe and compare survival across prognostic categories and disease trajectories. Patients for whom survival since prognosis was longer than a year were censored at day 400 in the Kaplan Meir curves. The log-rank test was used to compare survival between defined groups.

The characteristics of patients for whom prognosis was overestimated or underestimated were examined.

A sensitivity analysis excluding individuals who had a CMC record created after the start of the COVID-19 pandemic (March 2020) was carried out on the primary outcome i.e., the accuracy of the survival estimates to examine the hypothesis that prognostic accuracy may have been inflated, especially during the early months of the pandemic.

The study was reported using the RECORD (REporting of studies Conducted using Observational Routinely-collected Data) statement – extended from the STROBE (STrengthening the Reporting of OBservational studies in Epidemiology) statement (Supplementary Table 1).

## Results

One hundred eight thousand four hundred twenty-four eligible Coordinate My Care records were created during the study period. 10,237 (9%) records were excluded from the analysis of accuracy because they were missing information on prognosis prediction. The total analysed sample comprised 98,187 individuals (Table 1). During the observed period, 49,940 (51%) of the study cohort had died and 48,247 (49%) were alive at the time of the extract. Fifty seven percent of the cohort was female and 58% older than 80 years. Cancer and dementia/frailty were the most common diagnoses, 28% and 21% respectively. Cancer (43%) and dementia/frailty (21%) were also the most common diseases at the time of death. Most of the study population were frail (42% had WHO performance status 4, indicating the individual is incapable of performing any self-care). For those that had a place of death recorded, most died outside of hospital (79%). The preferred place of death and preferred place of care at the end of life were home and care home. Many patients had a recorded “do not attempt cardiopulmonary resuscitation” order (63%).

**Table 1:** Characteristics of the study sample containing prognostic estimates N (%)

	**Full prognosis cohort**	**Deceased**	**Alive**
	***n*** ** = 98,187**	***n*** ** = 49,940 (50.9%)**	***n*** ** = 48,247 (49.1%)**
**Gender** (*n* = 98,184)			
Female	56,397 (57.4%)	27,026 (54.1%)	29,371 (68. 9)
Male	41,787 (42.6%)	22,912 (45.9%)	18,875 (39.1%)
**Age Group at Record Creation** (*n* = 98,187)			
< 60	9,472 (19.2%)	4,177 (8.2%)	5,295 (11.0%)
60–69	10,556 (10.8%)	5,375 (10.8%)	5,181 (10.7%)
70–79	21,336 (21.7%)	10,315 (20.7%)	11,021 (22.8%)
≥ 80	56,883 (57.9%)	30,133 (60.3%)	26,750 (55.5%)
**Age Group at Death** (*n* = 98,125)			
< 60	3,966 (8.0%)	3,966 (8.0%)	-
60–69	5,175 (10.5%)	5,175 (10.5%)	-
70–79	9,907 (19.9%)	9,907 (19.9%)	-
≥ 80	30,830 (61.6%)	30,830 (61.6%)	-
**Diagnosis** (*n* = 98,096)			
Cancer	27,635 (28.1%)	21,444 (42.9%)	6,191 (12.8%)
Dementia & Frailty	20,475 (20.9%)	10,549 (21.1%)	9,926 (20.6%)
Heart Disease	14,965 (15.2%)	5,659 (11.3%)	9,306 (19.3%)
Respiratory Disease	7,926 (8.1%)	3,086 (6.3%)	4,840 (10.0%)
Other	27,095 (27.7%)	9,120 (18.4%)	17,975 (37.3%)
**WHO Performance Status** (*n* = 98,139)			
0 (Able to carry out all normal activity without restriction)	5,168 (5.3%)	406 (0.8%)	4,762 (9.9%)
1 (Restricted in strenuous activity but ambulatory and able to carry out light work)	7,232 (7.4%)	1,352 (2.7%)	5,880 (12.2%)
2 (Ambulatory and capable of all self-care but unable to carry out any work activities)	16,036 (16.3%)	5,054 (10.1%)	10,982 (22.8%)
3 (Symptomatic and in a chair or in bed for greater than 50% of the day but not bedridden)	28,135 (28.7%)	14,239 (28.5%)	13,896 (28.8%)
4 (Completely disabled; cannot carry out any self-care; totally confined to bed or chair)	41,568 (42.3%)	28,847 (57.9%)	12,721 (26.4%)
**Place of death** (*n* = 31,788)			
Home		12,714 (40.0%)	-
Care Home		8,110 (25.5%)	-
Hospice		4,030 (12.7%)	-
Hospital		6,821 (21.5%)	-
Other		113 (0.4%)	-
**Preferred Place of death – Option 1** (*n* = 66,694)			
Home	37,132 (53.3%)	22,105 (55.5%)	15,027 (50.4%)
Care Home	22,542 (32.3%)	12,904 (32.4%)	9,638 (32.3%)
Hospice	4,870 (7.0%)	3,456 (8.7%)	1,414 (4.7%)
Hospital	3,573 (5.1%)	745 (1.9%)	2,828 (9.5%)
Other	1,577 (2.3%)	650 (1.6%)	927 (3.1%)
**Preferred Place of death – Option 2** (*n* = 34,591)			
Home	9,593 (27.7%)	5,559 (28.3%)	4,034 (27.0%)
Care Home	7,218 (20.9%)	4,050 (20.6%)	3,168 (21.2%)
Hospice	10,526 (30.4%)	7,440 (37.8%)	3,086 (20.7%)
Hospital	6,021 (17.4%)	2,070 (10.5%)	3,951 (26.5%)
Other	1,233 (3.6%)	541 (2.8%)	692 (4.6%)
**Preferred place of care** (*n* = 83,645)			
Home	49,658 (59.4%)	29,298 (64.6%)	20,360 (53.2%)
Care Home	25,064 (30.0%)	14,048 (31.0%)	11,016 (28.8%)
Hospice	893 (1.1%)	601 (1.3%)	292 (0.8%)
Hospital	7,164 (8.6%)	1,134 (2.5%)	6,030 (15.8%)
Other	866 (1.0%)	295 (0.7%)	571 (1.5%)
**Presence of DNACPR order** (*n* = 98,187)			
Yes	61,798 (63.0%)	33,195 (66.5%)	28,603 (59.3%)
No	36,389 (37.0%)	16,745 (33.5%)	19,644 (40.7%)

*Abbreviations*: *WHO* World Health Organisation, *DNACPR* Do Not Attempt Cardiopulmonary Resuscitation. Patients who did not have resuscitation status recorded were included in the “For resuscitation” (No DNACPR order) group as this is a default treatment strategy in the UK

Out of a total cohort of individuals without a documented prognosis (n = 10,237), 81.5% had died at the time of data extraction. There were differences between these patients and the sample included in the analysis in terms of diagnosis, place of death and DNACPR status. In the cohort with missing prognosis information there was a slightly higher proportion of patients with cancer and dementia (34.4% versus 28.1% and 27.2% versus 20.9% respectively compared with the accuracy study cohort) and a lower proportion of patients with respiratory and cardiac disease. In the cohort with missing prognosis information, a much lower proportion died at home (29% versus 40%) and a higher proportion died in hospital (28.1% versus 21.5% in the cohort with prognostic information). There was a lower proportion of patients in the cohort without prognosis information with a DNACPR order in place (57.6% versus 63%) (Supplementary Table 2).

In the full prognosis cohort, 3% were predicted to live “days”; 13% “weeks”; 28% “months”; and 56% “year/years” (Table 2). The absolute agreement between prognostication and survival was 62% for the entire sample with an overall linear weighted Kappa of 0.634. Overall, clinicians “overestimated” prognosis (i.e., the patient died sooner than expected) in 23% of cases and underestimated prognosis (i.e., the patient lived longer than expected) in 15% of cases.

**Table 2:** Prognostication accuracy in the full cohort and stratified across major disease groups. Full prognosis cohort includes individuals with a primary diagnosis classified as “cancer”, “dementia/frailty”, “heart disease”, “respiratory disease” and “other”. Shaded cells for the “Prediction” and “Survival” denote those for whom the prediction was accurate. Weighted Kappa denotes the agreement between prognostication outcome and survival, the higher the value the better the prognostic ability. Q1 First quartile, the value in the dataset that holds 25% of the values below it. Q3 Third Quartile, the value in the dataset that holds 25% of the values above it

	**Survival (n, % of row total)**					**Median survival (Days)**	**Interquartile range(Days)**		**Weighted Kappa**
**Prognostic accuracy table—Full prognosis cohort (Overall 62% agreement between prediction and survival)**									
** Prediction**	*Days*	*Weeks*	*Months*	*Years*	***Σ (% grand total)***		*Q1*	*Q3*	0.634
* Days*	2,420 (74.2%)	412 (12.6%)	173 (5.3%)	255 (7.8%)	**3,260 (3.3%)**	4	2	13	
* Weeks*	4,415 (35.4%)	3,972 (31.9%)	2,364 (19.0%)	1,706 (13.7%)	**12,457 (12.7%)**	24	8	86	
* Months*	2,256 (8.3%)	5,976 (22.0%)	8,738 (32.2%)	10,153 (37.4%)	**27,123 (27.6%)**	146	41	535	
* Years*	585 (1.1%)	1,916 (3.5%)	6,926 (12.5%)	45,920 (83.0%)	**55,347 (56.4%)**	639	456	914	
*** Σ (% grand total)***	**9,676 (9.9%)**	**12,276 (12.5%)**	**18,201 (18.5%)**	**58,034 (59.1%)**	**98,187**				
**Prognostic accuracy table—Patients with Cancer**									
** Prediction**	*Days*	*Weeks*	*Months*	*Years*	***Σ (% grand total)***		*Q1*	*Q3*	0.725
* Days*	1,049 (79.5%)	184 (13.9%)	60 (4.5%)	27 (2.0%)	**1,320 (4.8%)**	4	2	11	
* Weeks*	2,500 (37.5%)	2,496 (37.5%)	1,207 (18.1%)	459 (6.9%)	**6,662 (24.1%)**	21	9	56	
* Months*	1,236 (9.6%)	3,860 (29.8%)	4,919 (38.0%)	2,919 (22.6%)	**12,934 (46.8%)**	83	32	239	
* Years*	101 (1.5%)	510 (7.6%)	1,544 (23.0%)	4,564 (67.9%)	**6,719 (24.3%)**	578	200	730	
***Σ (% grand total)***	**4,886 (17.7%)**	**7,050 (25.5%)**	**7,730 (28.0%)**	**7,969 (28.8%)**	**27,635**				
**Prognostic accuracy table—Patients with Dementia/Frailty**									
** Prediction**	*Days*	*Weeks*	*Months*	*Years*	***Σ (% grand total)***		*Q1*	*Q3*	0.749
* Days*	550 (74.9%)	67 (9.1%)	40 (5.4%)	77 (10.5%)	**734 (3.6%)**	4	2	13	
* Weeks*	866 (38.4%)	449 (19.9%)	402 (17.8%)	541 (24.0%)	**2,258 (11.0%)**	28	7	261	
* Months*	463 (8.4%)	690 (12.5%)	1,374 (24.9%)	2,987 (54.1%)	**5,514 (26.9%)**	395	80	670	
* Years*	206 (1.7%)	502 (4.2%)	1,856 (15.5%)	9,405 (78.6%)	**11,969 (58.5%)**	609	395	761	
***Σ (% grand total)***	**2,085 (10.2%)**	**1,708 (8.3%)**	**3,672 (17.9%)**	**13,000 (63.5%)**	**20,475**				
**Prognostic accuracy table—Patients with Heart Disease**									
** Prediction**	*Days*	*Weeks*	*Months*	*Years*	***Σ (% grand total)***		*Q1*	*Q3*	0.666
* Days*	247 (69.2%)	48 (13.4%)	19 (5.3%)	43 (12.0%)	**357 (2.4%)**	4	1	20	
* Weeks*	408 (36.2%)	267 (23.7%)	231 (20.5%)	220 (19.5%)	**1,126 (7.5%)**	30	7	174	
* Months*	250 (9.5%)	370 (14.1%)	709 (26.9%)	1302 (49.5%)	**2,631 (17.6%)**	308	61	639	
* Years*	114 (1.1%)	291 (2.7%)	1096 (10.1%)	9350 (86.2%)	**10,851 (72.5%)**	639	517	1064	
***Σ (% grand total)***	**1,019 (6.8%)**	**976 (6.5%)**	**2,056 (13.7%)**	**10,914 (72.9%)**	**14,965**				
**Prognostic accuracy table—Patients with Respiratory disease**									
** Prediction**	*Days*	*Weeks*	*Months*	*Years*	***Σ (% grand total))***		*Q1*	*Q3*	0.660
* Days*	122 (63.5%)	23 (12.0%)	12 (6.3%)	35 (18.2%)	**192 (2.4%)**	5	1	34	
* Weeks*	201 (35.1%)	132 (23.1%)	112 (19.6%)	127 (22.2%)	**572 (7.2%)**	34	8	234	
* Months*	139 (8.2%)	293 (17.4%)	526 (31.2%)	728 (43.2%)	**1,686 (21.3%)**	220	54	609	
* Years*	44 (0.8%)	138 (2.5%)	630 (11.5%)	4,664 (85.2%)	**5,476 (69.1%)**	639	517	806	
***Σ (% grand total)***	**506 (6.4%)**	**586 (7.4%)**	**1,280 (16.1%)**	**5,554 (70.1%)**	**7,926**				

The agreement between estimated and actual prognosis using linear weighted Kappa statistic was substantial for all disease groups and highest for patients with dementia/frailty (0.75) and cancer (0.73).The relatively good overall agreement between prognostic estimates and survival was influenced by the high accuracy of estimates that patients were likely to die within days (74%) or to live for more than a year (83%). The absolute agreement between prognostic estimates and survival was highest for patients expected to live “days” (74% in full cohort) or “year/years” (83% in full cohort) and this was mirrored in all disease categories. On the other hand, across all disease groups, clinicians were less accurate at predicting who would live for weeks or months (32% in both categories in the full cohort).

**Fig. 1:**
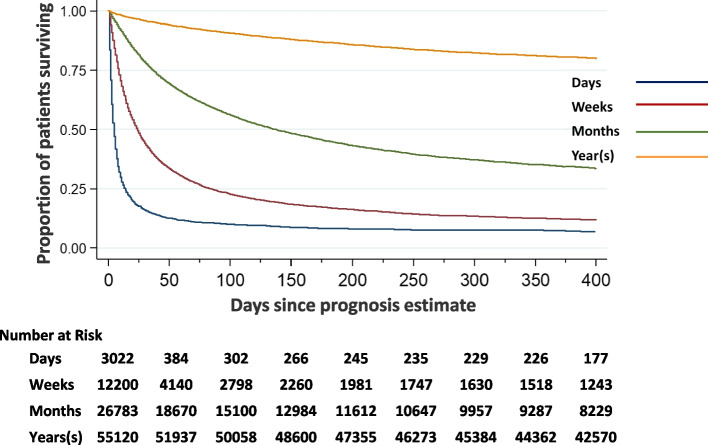
Kaplan–Meier survival curves for the full cohort across different prognostication categories. Notes: Survival is censored at day 400. Log ran test *p*<0.001

**Fig. 2:**
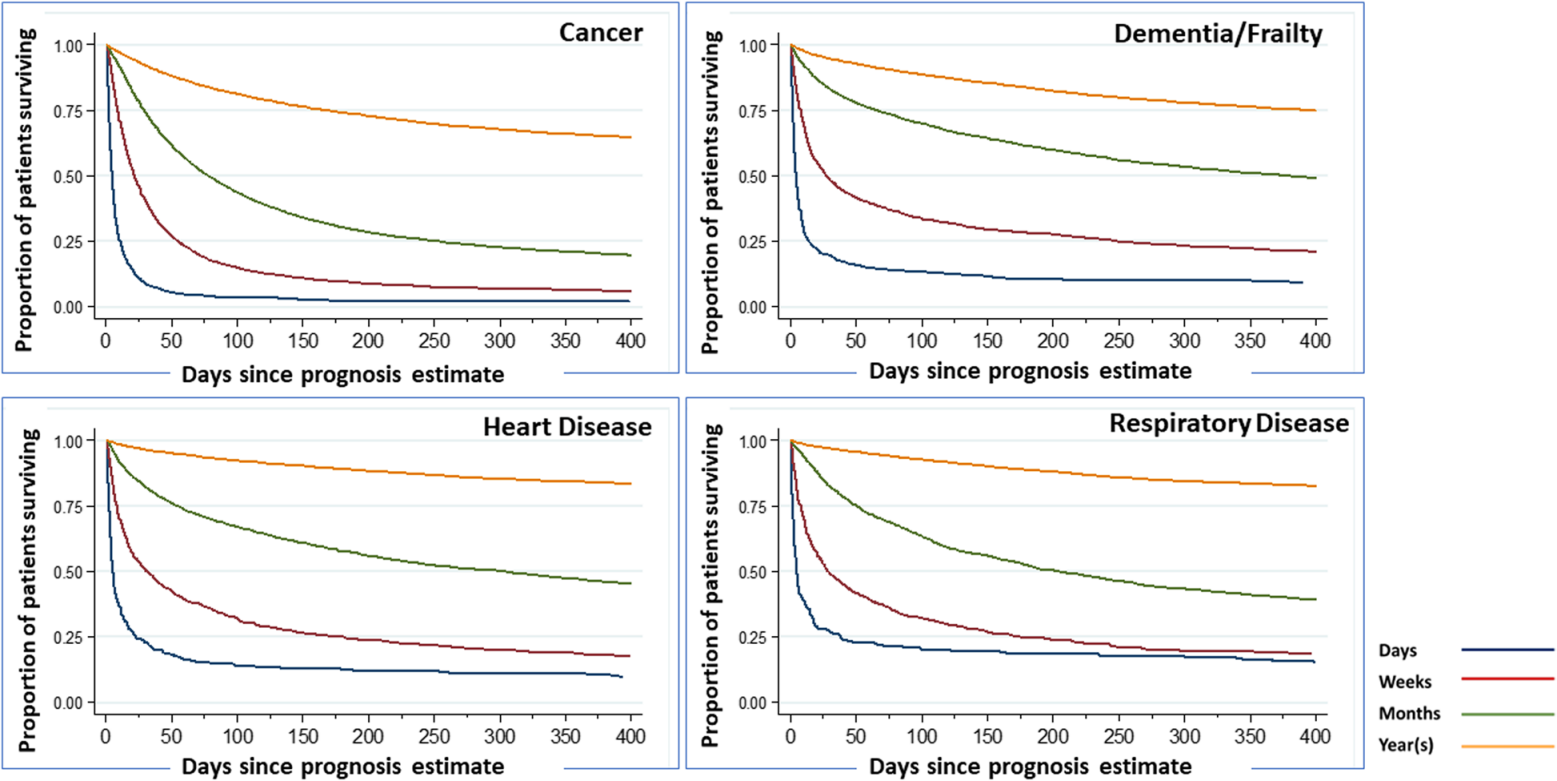
Kaplan–Meier survival curves for the major disease groups across different prognostication categories. Notes: Survival is censored at day 400. Log rank test for each disease group *p*<0.001. Additional data including numbers at risk are included in Additional File 2

Clinicians’ estimates were able to discriminate (log-rank *p* < 0.001) between groups of patients with differing survival prospects as illustrated by the Kaplan–Meier survival curves for the general cohort (Fig. 1). The median survival for each group fell within the expected upper and lower boundary limits. That is: patients expected to survive for “days” (< 14 days) survived for a median of 4 days; those expected to survive for “weeks” (≥ 14 days and < 60 days) survived for a median of 24 days; those expected to survive for “months” (≥ 60 days and < 365 days) survived for a median of 146 days, and those expected to survive for “years” (≥ 365 days) survived for a median of 639 days. Similar patterns were observed across all disease groups (Fig. 2).

There was no difference in the accuracy of prognostic estimates when individuals who had a CMC record created after the start of the COVID-19 pandemic (March 2020) were excluded (Supplementary Table 3).

A higher proportion of patients for whom providers overestimated prognosis were older, female, with a diagnosis of dementia and of those who died, a higher proportion died in a care home, as compared to those for whom providers underestimated prognosis (Supplementary Table 4).

## Discussion

### Main findings/results of the study:

Using a large set of routinely collected data we evaluated the accuracy of temporal predictions of survival in patients with a variety of advanced illnesses.

Our analysis demonstrates that the overall clinical accuracy of categorising patients into broad prognostic groups was 62%, mirroring prognostication estimates in other clinical studies [9, 14]. Clinicians were particularly good at identifying patients who were likely to live for fewer than 14 days. In a recent large multi-centre study of 1896 patients with cancer admitted to a palliative care unit, as in our study, survival estimates (using clinician’s predictions of survival or prognostic scales) were most accurate in patients with days to live. In that study however, all methods of prognostication demonstrated good performance for up to 60 days of survival, which is likely explained by the underlying diagnosis and the short overall median survival time (19 days), suggesting that patients were likely to have had more predictable trajectories [26]. In our study of patients with a variety of diagnoses and likely disease trajectories, clinicians were also good at identifying patients who were likely to live for more than one year but were less accurate at predicting survival of “weeks” or “months”. The greatest extent of agreement between estimated and actual prognosis was found for patients with dementia/frailty or cancer. The median survival of patients predicted to live for days, weeks, months, or years, fell within the expected range for the whole cohort and each disease category.

### What this study adds:

In this study, the accuracy of predictions about imminent death (within two weeks) was 74.2% across the full patient cohort. The best accuracy for this time frame was in cancer patients (79.5%) and the worst was in patients with respiratory disease (63.5%). Our findings align with the more typically predictable short period of rapid decline which is often described in patients with cancer, as compared to the gradual and progressive deterioration in patients with other diagnoses [20, 27, 28]. For patients with heart or lung disease, frequent exacerbations in their clinical condition on the background of a gradual decline in health and functional status make identification of the dying phase more challenging [20]. Nonetheless, our results provide reassurance that for the majority of patients, regardless of diagnosis, clinicians are reasonably accurate at identifying the last few days of life.

Clinicians were also good at recognising which patients were likely to live for more or less than one year. Of the 55,347 patients predicted to survive for more than one year, 45,920 (83%) did so. Conversely, of the 39,840 people predicted to live for less than one year, 30,726 (77%) did so.

This study demonstrates the significant difference in the accuracy of survival estimates depending on the prognostic category. Factors that influence the accuracy of survival estimates are yet to be defined but are likely to be complex and multiple [18]. Whilst prognostic models vary in levels of sophistication [29], despite its central importance, clinicians are rarely trained for prognostication [30]. Clinicians usually consider a number of patients’ characteristics when providing a prognosis, such as performance status, underlying diagnosis, clinical observations, symptoms, comorbidities and the known overall disease trajectory [24, 31–33]. Clinicians also often rely on their own clinical experience and intuition. This may, however, be subject to explicit or implicit cognitive bias [17]. Clinicians acknowledge the challenges of estimating survival and discussing estimates with patients and families [23]. In addition, there is an inherent human desire to preserve hope, which may consciously or unconsciously affect clinicians’ communications about prognosis. Timing of prognosis is relevant, as evidence suggests that survival estimates may become more accurate when people are nearing end-of-life, a phenomenon known as “the horizon effect” [1, 34]. This finding is mirrored in the data presented in this study. However, survival predictions remain a challenge for many clinicians, even for patients who are imminently dying [35].

Our study shows that it is more difficult to identify patients who will die within “weeks” or “months” than “days” or “years”. Although many of these patients died within or earlier than the upper bound of the predicted period (e.g., most patients predicted to die within “weeks” died in fewer than 60 days [8,387/12,457 = 67%]), these data highlight the significant prognostic uncertainty faced by patients, who are neither imminently dying nor expected to live for “years”. Approaches and interventions to support patients whose situations are clinical uncertain assume greater importance [36–38] greatly amplified during the COVID-19 pandemic [39]. Advance care planning (i.e., enabling patients to define goals and preferences for future medical treatment and care, and to record and share these discussions and decisions with relevant health and social care providers) [40] attempts to ensure that care is patient-centred even when their situation is uncertain. Early palliative care may also have a role here. The introduction of palliative care early in a patient’s disease journey has been shown to support communication and improve quality of life [3, 41, 42].

The majority of the records contained within this Electronic Palliative Care Coordination System included a prognostic estimate, which is reflective of the nature of such systems. However, in our cohort, patients with missing information on prognosis were much more likely to die in hospital and much less likely to die at home, despite the finding that a similar proportion wanted to die either at home or in a care home. Sensitive and timely discussions around prognosis may be a helpful part of the process of advance care planning for some patients, acknowledging that this is not something that every patient wants to engage with.

In this study, we observed high levels of prognostic accuracy for patients with dementia who were expected to die within days and for those with a life expectancy of year/years but poor accuracy of survival estimates of “weeks” or “months”. Patients with dementia however often experience an unpredictable disease trajectory. This can make identification of the end-of-life and prognostication challenging [43]. Our data support international recommendations that the initiation of palliative care for patients with dementia should be based on need rather than prognostication [44].

### Strengths and weaknesses/limitations of the study:

This was a large study involving patients with varying diagnoses. As a result, we were able to make robust estimates of the accuracy of survival for different groups of patients and evaluate prognostic accuracy. These data provide a real-life insight into survival predictions incorporated into individual patients’ clinical care decisions and care preferences. This study was large enough to enable stratification and comparison across major disease trajectories, facilitating detailed insight into disease-specific prognostication. Having a temporal estimate of survival as a mandatory data field ensured that prognosis information was available for 91% of the overall cohort in the observed period.

Our analyses were limited due to the format of the available data. The survival prediction was based on categorical values, consequently we are unable to calculate the area under the receiver operating characteristics curve (AUROC). Linear weighted kappa coefficients were used to determine “true” agreement between estimates and actual survival, considering the agreement that would be expected by chance [45] and giving an average of the kappa coefficients across the different survival categories [46]. Our use of LWK and percentage of accurate estimates supports comparison with other prognostic studies in which this approach was used [9, 18].

Patients with a Coordinate My Care record are expected to have a limited life expectancy as the service is designed for patients with advanced conditions and therefore our results cannot be generalised to other patient cohorts. This cohort was based in London, so results may not be generalisable to non-urban settings. Although the role of the clinician in documenting prognosis in Coordinate My Care is recorded, it is not possible to determine which health care professional made the prognosis or the role of the multi-disciplinary team in this process [47]. A previous systematic review concluded that there was no difference in the accuracy of prognostic estimates between clinician sub-groups [18]. Prognostic estimates may be revised over time, and subsequent estimates may be more or less accurate than initial ones. We did not include longitudinal changes in prognostication in our analysis. Our data included some Coordinate My Care records that were created during the initial months of the COVID-19 pandemic. It is possible that the onset of a new and unknown disease made accurate prognoses even more difficult, Our data did not however demonstrate any difference in prognostic accuracy when prognostic estimates from records created after the start of the pandemic were included.

Our study was based on the retrospective analysis of routinely collected data and does not give any information about the information needs of individual patients about prognostic estimates.

## Conclusion

Prognosticating in clinical practice is challenging, however, our results suggest that clinician predictions of survival are generally good at identifying individuals at either end of the survival spectrum (i.e., those who will die imminently and those who will live for much longer). The accuracy of prognostication for these time frames differs across major disease categories, but remains acceptable even in non-cancer patients, including those with dementia. Identifying patients who will live more than “days” but less than “months”, or more than “weeks” but less than “years” is more challenging and further research to improve prognostic accuracy for these patients is needed. For these patients with more uncertain outcomes, advance care planning and early introduction to palliative care may be particularly beneficial. We suggest that training for clinicians in having sensitive discussions around prognosis [33], should include an acknowledgement of the significant level of prognostic uncertainty for patients who are not thought to be imminently dying with an emphasis on the need for appropriate and individualised support according to how much information the patient and their family wants to know [48].

## Supplementary Information

**Additional file 1:Supplementary Table 1.** The RECORD statement – checklist of items,extended from the STROBE statement, that should be reported in observationalstudies using routinely collected health data. *Reference: Benchimol EI, SmeethL, Guttmann A, Harron K, Moher D, Petersen I, SørensenHT, von Elm E, Langan SM, the RECORD Working Committee.  The REporting of studies Conducted usingObservational Routinely-collected health Data (RECORD) Statement.  *PLoSMedicine *2015. **SupplementaryTable 2.** Patients without arecorded prognostic estimate. **Supplementary Table 3.** Prognostic accuracyexcluding records created after start of COVID19 pandemic (March 2020). **SupplementaryTable 4.** Patients with over and underestimated prognosis.

**Additional file 2.**

## Data Availability

Anonymised data from this study are stored on the Biomedical Research Informatics Digital Environment (BRIDgE), a Trusted Research Environment (TRE) and informatics platform at The Royal Marsden Biomedical Research Centre. Data sharing requests and access to the protocol and supplementary information would be available on reasonable request after completion of existing studies and whenever legally and ethically possible. Data access requests should be directed to Dr Joanne Droney joanne.droney@rmh.nhs.uk. Access requests will be reviewed and authorised by The Royal Marsden Committee for Clinical Research and Information Governance Committee.
